# Ubiquitin-specific protease 7 downregulation suppresses breast cancer in vitro

**DOI:** 10.3906/biy-1912-83

**Published:** 2020-08-19

**Authors:** Taha Bartu HAYAL, Ayşegül DOĞAN, Hatice Burcu ŞİŞLİ, Binnur KIRATLI, Fikrettin ŞAHİN

**Affiliations:** 1 Yeditepe University, Department of Genetics and Bioengineering, Faculty of Engineering, İstanbul Turkey

**Keywords:** Ubiquitination, breast cancer, USP7, p5091, gene editing

## Abstract

Because breast cancer is complicated at the pathological, histological, clinical, and molecular levels, identification of new genetic targets against carcinogenic pathways is required to generate clinically relevant treatment options. In the current study, ubiquitin-specific protease 7 (USP7), which regulates various cellular pathways including Mdm2, p53, and NF–κB, was selected as a potential gene editing strategy for breast cancer in vitro. Anticancer activity of USP7 gene suppression has been evaluated through cell proliferation, gene expression, cell cycle, sphere dissemination, and cell migration analysis. Here, siRNA and shRNA strategies and an allosteric small-molecule inhibitor of USP7 were used to define potential anticancer activity against MCF7 and T47D human breast cancer cell lines. Both blockage of deubiquitination by p5091 and knockdown of USP7 reduced cell proliferation, cell migration, colony formation, and sphere dissemination for both MCF7 and T47D breast cancer cell lines. Restriction of USP7 activity strongly enhanced apoptotic gene expression and reduced metastatic ability of breast cancer cell lines. This study describes one potential molecular target for the suppression of breast cancer proliferation and metastasis. Identification of USP7 as a promising gene editing candidate might open up the possibility of new molecular drug research in targeting the ubiquitination pathway in cancer.

## 1. Introduction

Breast cancer is abnormal cell proliferation in the ductal and gland regions of the breast tissue. Breast cancer is the most common cancer among women and accounts for 30% of all newly diagnosed cases of cancer in the female population. The estimated number of breast cancer patients and deaths by the end of 2020 in the United States will be approximately 279,100 and 42,690 respectively (Siegel et al., 2020). Treatment options for breast cancer are various and challenging due to the complex nature of the disease and the different subtypes with diverse biological properties and metastatic ability (Lyden et al., 2011). Treatment strategy is based on the cancer stage, aggressiveness, cell surface (HER2), and steroid hormone receptors expression (PR and ER) profile, which affect mortality (Al-Mahmood et al., 2018). Breast cancer is a multistep process regulated by the activation and suppression of several molecular pathways which control the cellular behavior and carcinogenesis process at the molecular level. Identification of molecular mechanisms that are responsible for breast cancer development is important in order to generate more specific treatments. Breast cancer cell lines MCF7 and T47D, which are both classified as Luminal A (ERα+, PR+/–, and HER2–) (Yu et al., 2017), differ in p53 mutation status. The MCF7 cell line has a wild-type p53 protein (Lim et al., 2009), while T47D has a L194F mutation in its p53 protein (Parrales and Iwakuma, 2015). MCF7 and T47D cell lines were used to compare the effect of USP7 with the p53 protein pathway as approximately half of cancer patients carry the mutant p53 protein (Gasco et al., 2002), which has been correlated with poor prognosis in breast cancer patients (Lai et al., 2004).

Ubiquitination is one of the main mediators of posttranslational protein modifications that control major breast cancer pathways (Ohta and Fukuda, 2004). Although previous studies have mainly focused on phosphorylation as a posttranslational modification, recent studies have proved that ubiquitination has a crucial role in various signaling and cell regulatory pathways in breast cancer (Pal and Donato, 2014). Ubiquitination and deubiquitination of proteins control various cellular mechanisms such as cell cycle progression (King et al., 1996) and regulation of transcription factors (Ciechanover, 2013). The ubiquitin proteasome pathway not only degrades proteins but also controls the carcinogenesis process at the molecular level, including DNA repair mechanisms (Gallo et al., 2017). The activity of ubiquitin ligases and deubiquitinase enzymes maintains the oncogenic pathways (Gallo et al., 2017) which might control cancer initiation, progression, or promotion. Several regulatory pathways, such as the negative feedback mechanism of p53 protein, are controlled by the ubiquitination process in breast cancer (Mourtzoukou et al., 2018).

USP7 is a member of the ubiquitin specific protease (USP) deubiquitination family; it interacts with many other cellular proteins (Sowa et al., 2009). USP7 recycles associated proteins and prevents proteasomal degradation via deubiquitination. USP7 interacts with several proteins that are involved in the cell cycle, DNA repair, and epigenetic mechanisms. Overexpression of USP7 is observed in many types of cancers, indicating the therapeutic potential of USP7 as a target (Wang, 2019). Aberrant overexpression of USP7 has been observed in breast cancer (Wang et al., 2016).

Identification of the ubiquitination pathways that control breast cancer initiation, promotion, or progression might be useful for clinics investigating new treatment modalities. Development of gene editing techniques might enable targeting strategies against ubiquitination pathways to treat breast cancer. The current study aims to investigate the promoting role of USP7 in breast cancer cell lines in vitro using small-molecule–based inhibition and siRNA/shRNA-based knockdown strategies to downregulate USP7 in MCF7 and T47D cell lines in vitro.

## 2. Materials and methods

### 2.1. Cell lines 

T47D (HTB-133), MCF7 (HTB-22), and HEK293T (CRL-3216) cell lines were provided from the American Type Culture Collection (ATCC, Rockville, MD, USA). T47D cells were incubated in Roswell Park Memorial Institute medium (RPMI, Gibco, Paisley, Scotland, UK); MCF7 and HEK293T cells were incubated in Dulbecco’s Modified Eagle’s Medium (DMEM, Gibco) supplemented with 10% fetal bovine serum (FBS, Gibco) and 1% penicillin/streptomycin/amphotericin (PSA, Gibco). Cells were incubated in a humidified incubator at 37 °C and 5% CO2. 

### 2.2. Small molecule inhibitor preparation 

DUB inhibitor (p5091, Selleckchem, Houston, TX, USA) was prepared in dimethyl sulfoxide (DMSO, Sigma-Aldrich Chemie GmbH, Hamburg, Germany) as described in the literature (Tavana et al., 2018); 10 mM stock concentration was prepared in DMSO, and further concentrations were prepared in complete cell culture medium.

### 2.3. siUSP7 transfection 

USP7-specific small interfering RNA was purchased from ThermoFisher Scientific (siRNA ID: 105065). Transfection protocol was performed using Lipofectamine® RNAiMAX (Thermo Fisher Scientific Inc., Invitrogen, Carlsbad, CA, USA) according to the described protocol. Briefly, cells were seeded onto 12-well plates (5 × 104 cells/well) prior to transfection and incubated until 80% confluence. Cells were treated with 20 pmol of silencer siRNA negative control (Thermo Fisher) and USP7 siRNAs for 48 h and subjected to further experiments. 

### 2.4. Cell viability assay 

Effect of DUB inhibitor p5091 on cell viability of T47D and MCF7 cells was measured with 3-(4,5-dimethyl-thiazol-2-yl)-5-(3-carboxy-methoxy-phenyl)-2-(4-sulfo-phenyl)-2H-tetrazolium (MTS) assay (CellTiter96 AqueousOne Solution; Promega UK Ltd., Southampton, UK). Briefly, cells were seeded on 96-well plates (Corning Inc., Corning, NY, USA) at a cell density of 5 × 103 cells/well. Cells were treated with various concentrations of p5091 (2μM, 5μM, 10μM, and 20μM) for 24, 48, and 72 h. In order to measure cell viability, cells were exposed to PBS containing 4.5 g/L glucose and 10% MTS as described previously (Huang et al., 2004). Absorbance rates at 490 nm, indicating viability, were measured after 1–2 h incubation of cells in a humidified incubator in darkness.

### 2.5. Quantitative real-time PCR (qPCR) assay

Specific primers for ubiquitin-specific protease 7 (USP7), glyceraldehyde 3-phosphate dehydrogenase (GAPDH), protein kinase B (Akt), Bcl–2-associated X protein (BAX), B-cell lymphoma 2 (BCL2), caspase 3, caspase 7, Forkhead box protein O4 (FOXO4), epidermal growth factor receptor (EGFR), nuclear factor kappa B (NF-κB), and mouse double minute 2 homolog (MDM2) (Table) were designed with Primer-BLAST software (National Center for Biotechnology, Bethesda, MD, USA) and synthesized by Macrogen Inc. (Seoul, Korea). The SYBR Green method was used to detect mRNA levels of the target genes (Navarro et al., 2015). GAPDH was used as a housekeeping gene. RNA was isolated using TRIZOL (Invitrogen) according to the manufacturer’s instructions and cDNA was synthesized using an iScript™ cDNA Synthesis Kit (Bio-Rad Laboratories Inc., Hercules, CA, USA) and qPCR assay was performed by using a CFX96 RT-PCR system (Bio–Rad Laboratories Inc.).

**Table:** qPCR primer sequences.

Gene	Side	Sequence
USP7	Forward	5’ GGAAGCGGGAGATACAGATGA 3’
	Reverse	5’ AAGGACCGACTCACTCAGTCT 3’
MDM2	Forward	5’ GGCTCTGTGTGTAATAAGGGAGA 3’
	Reverse	5’ GGACTGCCAGGACTAGACTTTG 3’
P53	Forward	5’ GCCCAACAACACCAGCTCCT 3’
	Reverse	5’ CCTGGGCATCCTTGAGTTCC 3’
BAX	Forward	5’ TGCAGAGGATGATTGCCGCCG 3’
	Reverse	5’ ACCCAACCACCCTGGTGTTGG 3’
BCL2	Forward	5’ AACGGAGGCTGGGATGCCTTTGTG 3’
	Reverse	5’ ACCAGGGCCAAACTGAGCAGAGT 3’
Caspase 3	Forward	5’ GAGGCGGTTGTAGAAGAGTTCGTG 3’
	Reverse	5’ TGGGGGAAGAGGCAGGTGCA 3’
Caspase 7	Forward	5’ GGAGAAAGCTCATGGCTGTGT 3’
	Reverse	5’ TCCCCTTGGCTGTGTTTTG 3’
EGFR	Forward	5’ AATGCAACATCCTGGAGGGG 3’
	Reverse	5’ AGGTGATGTTCATGGCCTGG 3’
PTEN	Forward	5’ TGTGGTCTGCCAGCTAAAGG 3’
	Reverse	5’ ACACACAGGTAACGGCTGAG 3’
FOXO4	Forward	5’ GACTGCGAGTCCATCATCCT 3’
	Reverse	5’ GGGCTGAGTCGAAGTTGAAG 3’
Akt	Forward	5’ GAAGCTGCTGGGCAAGGGGCA 3’
	Reverse	5’ GTGGGCCACCTCGTCCTTGG 3’
NF - κB	Forward	5’ GCCACCCGGCTTCAGAATGGC 3’
	Reverse	5’ TATGGGCCATCTGCTGTTGGCAGT 3’
GAPDH	Forward	5’ AAGGTGAAGGTCGGAGTCAAC 3’
	Reverse	5’ GGGGTCATTGATGGCAACAATA 3’

### 2.6. Western blot analysis

Change in USP7 protein expression and total ubiquitination were determined by Western blot analysis. Total protein was isolated by RIPA Lysis buffer; 30 µg proteins were loaded on MiniPROTEAN Precast Gel (Bio-Rad Laboratories Inc.) and transferred to PVDF membrane (Bio-Rad Laboratories Inc.). Membranes were blocked with TBST containing 5% Blotting Grade Blocker (Bio-Rad Laboratories Inc.) for 1 h. USP7-specific antibody (Cell Signaling Technology, Inc., Danvers, MA, USA) or mono- and poly-ubiquitinated conjugates antibody (Enzo Life Sciences GmbH, Lörrach, Germany) was applied in blocking solution overnight (4 °C). Membrane was incubated with antirabbit secondary antibody (dilution 1:5000, Santa Cruz Biotechnology Inc., Dallas, TX, USA) for 2 h at room temperature and incubated with Amersham ECL™ Detection Reagents (Sigma-Aldrich Chemie GmbH) for 30 s. GAPDH was used as an internal control and images were taken by using the luminometer system (Bio-Rad Laboratories Inc.). 

### 2.7. Colony forming unit (CFU) assay

Colony forming capability of the cells was determined via CFU assay according to previously described protocol (Digirolamo et al., 1999). T47D and MCF7 cell lines were plated in 6-well plates (Corning Plasticware) at a cell density of 300 cells/well in complete culture medium. After 24 h, cells were treated with 10 µM p5091 and siUSP7. After 2 days for MCF7 and 3 days for T47D, media were changed to growth medium and cells were incubated for 14 days until colony formation could be observed. Cells were fixed with 4% paraformaldehyde and stained with crystal violet. Images were taken with a ZEISS microscope equipped with an AxioCam ICc 5 camera and using ZEN 2 (Blue edition) software.

### 2.8. Cell cycle analysis

In order to determine the effect of USP7 suppression on the control of cell division, T47D and MCF7 cells were subjected to cell cycle analysis according to the protocol (Demirci et al., 2019). Briefly, cells were plated (3 × 105 cells/well) in 6-well tissue culture plates; after 24 h incubation, siRNA or p5091 was applied to the cells. Cells were fixed with 4% paraformaldehyde and suspended in 500 μL staining solution containing 40 μg/mL RNase A (Fisher Scientific UK Ltd., Loughborough, UK), 33 μg/mL propidium iodide (PI, Fisher Scientific UK Ltd., Loughborough, UK) and 0.2% nonyl phenoxypolyethoxylethanol [NP -40, Fisher Scientific UK Ltd., Loughborough, UK) in PBS]. Cells were incubated in staining solution at 37 °C for 30 min, and the cell cycle was analyzed using BD FACS Calibur (BD Biosciences, Woburn, MA, USA).

### 2.9. Hanging drop assay 

Sphere formation capability of breast cancer cells was determined by hanging drop assay to detect whether USP7 suppression might block sphere formation in vitro (Kuo et al., 2017). Cells were counted and diluted to 1 × 103 cells per 40 μL of media containing 10 μL of 0.1% gelatin after p5091 and siUSP7 administration. Cell and gelatin were mixed well and transferred drop by drop to the lid of a 100-mm culture dish (Corning Plasticware). Each 40-μL volume drop had an approximate diameter of 1.4 mm. An appropriate amount of PBS was added into the culture dish to minimize evaporation. The culture vessel lid with the cell drops was gently flipped upside down to create the gravitational force required for sphere formation. After 24 h, spheres formed and were transferred into cell-culture–compatible 24-well plates for examination under the light microscope. Sphere diameter and thus sphere dissemination were checked for in the following 2 days. Images were taken with a ZEISS microscope equipped with AxioCam ICc 5 camera and ZEN 2 (Blue edition) software.

### 2.10. Scratch assay

To identify the changes in metastatic capacity, scratch assay protocol was performed as described in the literature (Doğan et al., 2014). Briefly, cells were seeded on 12-well plate dishes (Corning Plasticware) as 105 cells/well in the appropriate medium for each cell; they were allowed to attach in a humidified 37 °C and 5% CO2-conditioned incubator. The attachment of the cells was followed by scratching a wound with a sterile 200-μL pipette tip. Deattached cells were washed once with 1× PBS solution followed by fresh medium addition, which included 10µM p5091 or 20 pmol siUSP7. Images were taken via ZEISS microscope, AxioCam ICc 5 camera, and the ZEN 2 (Blue edition) computer application.

### 2.11. USP7 knockdown using shRNA

In order to investigate the effect of stable USP7 knockdown on colony-forming capacity, USP7 protein was knocked down with lentiviral shUSP7 vector (V3SH11252, Dharmacon Inc., Cambridge, UK) according to the procedure in the literature (Mali, 2013). 

HEK293T cell line was transfected with the combination of shUSP7 vector, lentiviral envelope plasmid [pCI-VSVG (Addgene, MA, USA)], and packaging plasmid (pCMV-dR8.2 dvpr [Addgene]) for lentiviral production in the collected medium was examined with Lenti-x Go Sticks (Takara Bio Inc., Shiga, Japan), according to the manufacturer’s instructions. For transduction, MCF7 and T47D cells were cultured into 24-well plates (Corning Plasticware) 24 h before viral infection at a density of 5 × 104 cells per well. After overnight incubation, the culture medium was refreshed with 1 mL of fresh complete media containing viral particles (4 × 104 transducing units/mL) and 10 μg/mL polybrene (Sigma-Aldrich Chemie GmbH). Cells were incubated in a humidified incubator at 37 °C and 5% CO2 for 24 h. After observing GFP signals, transduced cells were selected in complete growth medium containing 2 μg/mL puromycin (Sigma-Aldrich Chemie GmbH). 

### 2.12. Statistical analysis

All experiments were conducted at least 3 times. Statistical analyses were conducted using one-way ANOVA and Tukey’s post hoc test using GraphPad Prism 7 software (GraphPad Software Inc., San Diego, CA, USA). Statistical significance was determined as * P < 0.05, ** P < 0.005, *** P < 0.001. 

## 3. Results

### 3.1. USP7 inhibition decreases cell viability 

**Figure 1:**
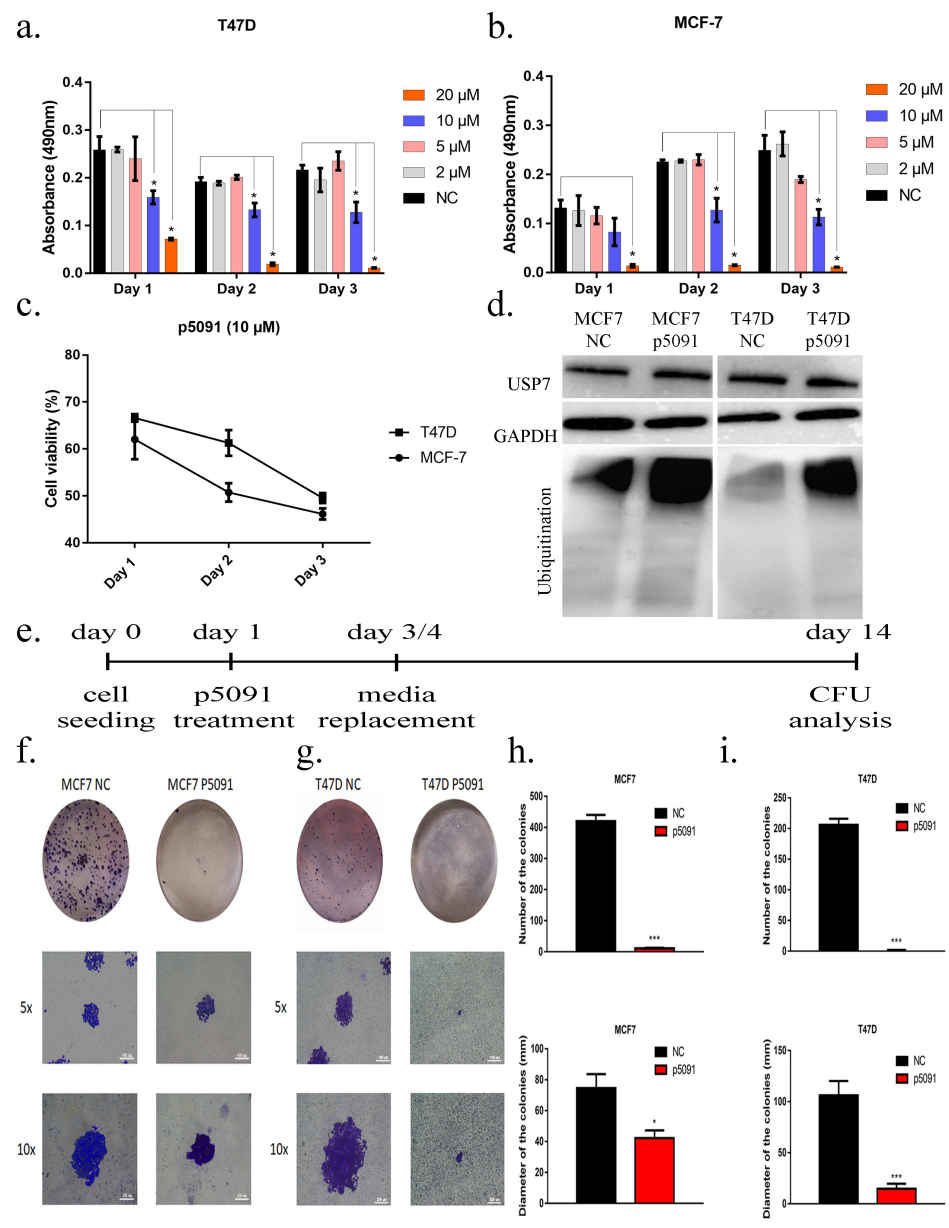
p5091 blocks cell proliferation and CFU formation in vitro. (a) Cell viability analysis of p5091 treated T47D cells. (b) Cell viability analysis of p5091 treated MCF7 cells. (c) Cell viability analysis of 10μM p5091 treated MCF7 and T47D cells. (d) Western blot analysis of MCF7 and T47D cells after 10μM p5091 administration. 30μg of total protein was blotted with anti-USP7 and antiubiquitin antibodies. Anti-GAPDH antibody was used for loading control. (e) Experimental design of CFU analysis after p5091 treatment. (f) Microscopic images of CFU assay after 10μM p5091 treatment for 48 h in MCF7 and (g) for 72 h in T47D. (h) Number of the colonies and diameter of the colonies after 48 h of p5091(10μM) treatment in MCF7 and (i) 72 h of p5091 (10μM) treatment in T47D cells. * P < 0.05, *** P < 0.001, scale bar: 400μm (5x), 200μm (10x). NC: Negative control, number of replicates: 3.

MTS assay was performed to understand the effect of USP7 inhibitor p5091 on the cell viability of breast cancer cell lines for 3 days. Different concentrations (2µM, 5µM, 10µM, and 20µM) of p5091 were tested on T47D and MCF7 cell lines. Results have shown that cell viability was reduced in a concentration-dependent manner, and 10µM p5091 caused an approximately 50% decrease in T47D (Figure 1a) and MCF7 (Figure 1b) cell viability after 3 and 2 days of treatment, respectively (Figure 1c). Small-molecule–inhibitor p5091 treatment had no effect on USP7 protein level in either MCF7 (after 48 h of treatment) or T47D (after 72 h of treatment) as expected, since p5091 reduces enzyme activity instead of decreasing the protein amount. However, total ubiquitination levels significantly increased (≈4 fold) (Figure 1d), indicating the inhibition of USP7 activity in vitro for the selected concentration of p5091 (10µM). CFU assay was performed to identify the alterations in colony-forming ability of the breast cancer cells after allosteric inhibition of USP7 (Figure 1e). The results demonstrated that blocking the activation of USP7 in MCF7 cells reduced the diameter of colonies approximately 45% compared to control (Figure 1f). Moreover, USP7 inactivation decreased the diameter of the colonies approximately 85% in cultured T47D cells (Figure 1g). The number of MCF-7 and T47D colonies dramatically decreased in p5091-treated cells. Although growth-medium–treated negative control cells formed 420 colonies, the p5091 group formed 11 colonies in MCF-7 cells (Figure 1h). Similar results were obtained in T47D cells as well; the number of colonies decreased from 212 to 9 (Figure 1i).

**Figure 2:**
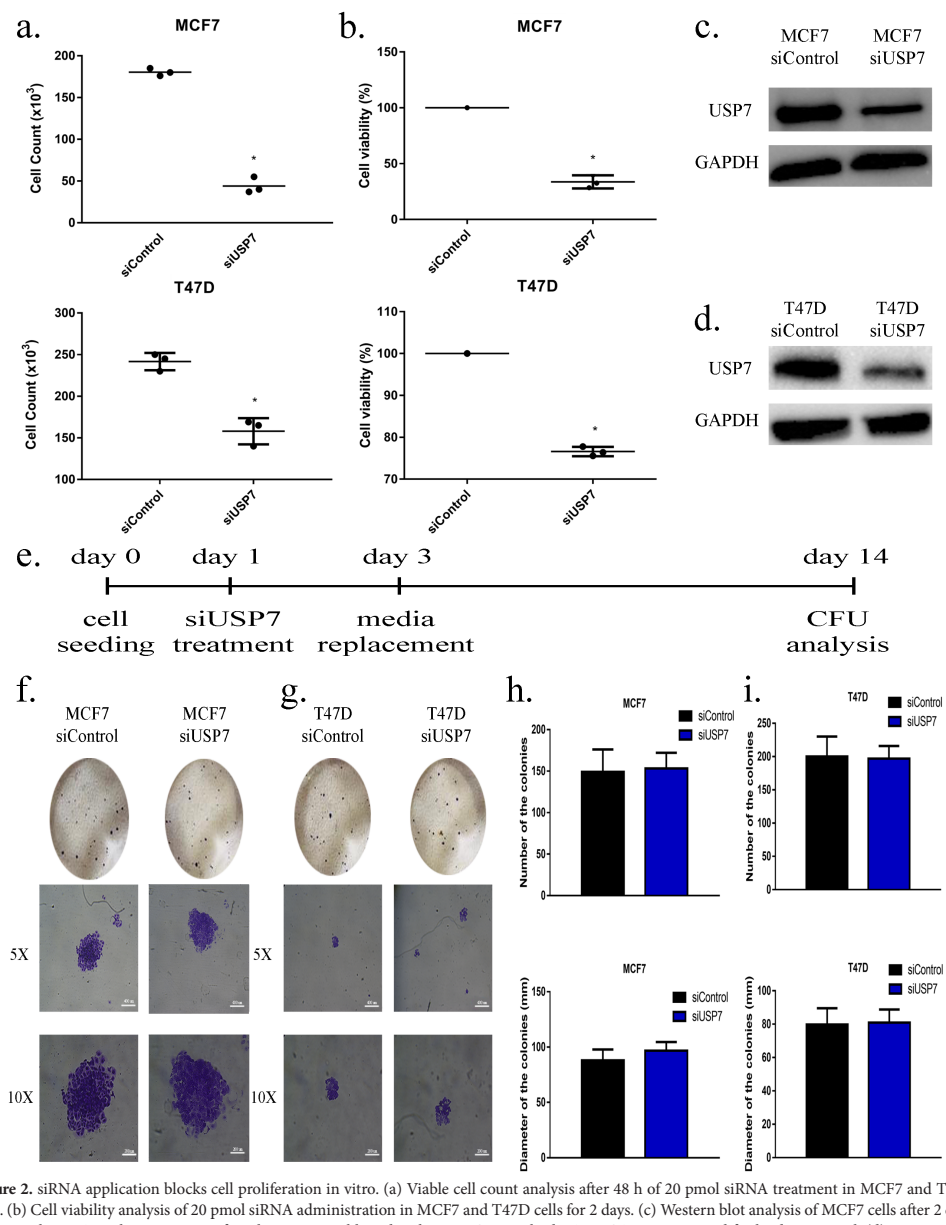
siRNA application blocks cell proliferation in vitro. (a) Viable cell count analysis after 48 h of 20 pmol siRNA treatment in MCF7 and T47D cells. (b) Cell viability analysis of 20 pmol siRNA administration in MCF7 and T47D cells for 2 days. (c) Western blot analysis of MCF7 cells after 2 days of 20 pmol siRNA application. 30μg of total protein was blotted with anti-USP7 antibody. Anti-GAPDH was used for loading control. (d) Western blot analysis of T47D cells after 2 days of 20 pmol siRNA application. 30μg of total protein was blotted with anti-USP7 antibody. Anti-GAPDH was used for loading control. (e) Experimental design of CFU analysis after siUSP7 application. Cells were treated with 20 pmol siRNA for 2 days and culture media was refreshed after every 48 h for 14 days. (f) Microscopic images of CFU assay after transient USP7 knockdown in MCF7 and (g) T47D cells. (h) Number of the colonies and diameter of the colonies after transient USP7 knockdown in MCF7 and (i) T47D cells. * P < 0.05, scale bar: 400μm (5x), 200μm (10x). siControl: Control small interfering RNA, siUSP7: USP7 small interfering RNA, number of replicates: 3.

Apart from the inhibition of USP7 activity via small-molecule inhibition, siRNA knockdown strategy was used to reduce USP7 at the molecular level to understand the regulatory role of USP7 on breast cancer cell proliferation and growth in vitro. Viable cell counts were calculated after siRNA treatment to observe cell proliferation followed by USP7 knockdown. siUSP7 treatment decreased the cell viability of MCF7 cells from 180 × 10^3^ living cells to 40 × 10^3^ cells (77.78% reduction) (Figure 2a). Similarly, the viable T47D cell number decreased from 245 × 10^3^ to 165 × 10^3^, a 32.65% decrease (Figure 2b). The efficacy of siUSP7 strategy was examined by Western blot analysis. USP7 protein levels decreased 5-fold and 3-fold for MCF7 (Figure 2c) and T47D (Figure 2d), respectively. Additionally, CFU assay was performed to examine the alterations in stemness capability after silencing USP7 protein level by specific siRNA (Figure 2e). However, there was no significant change in the morphology of MCF7 (Figure 2f) and T47D (Figure 2g) colonies. Similarly, no significant change was observed in CFU ability of MCF7 and T47D after siRNA treatment, indicating that the effect of siRNA-based gene downregulation of USP7 in MCF7 (Figure 2h) and T47D (Figure 2i) cells was transient. Furthermore, stable knockdown of USP7 was performed to prove that a loss of USP7 function decreases colony-forming capacity, but the regaining of USP7 enzyme activity can restore the stemness of cells (Supplementary 1). The successful transfection of shUSP7 and control vector (Scrambled) was shown via fluorescent microscopy images in MCF7 cell lines (Supplementary Figure 1a). USP7 protein level decreased by nearly 60% compared to GAPDH (Supplementary Figure 1b). Moreover, CFU assay in the MCF7 cell line (Supplementary Figure 1c) showed that stable knockdown of USP7 decreased colony numbers from 15 to 3 (Supplementary Figure 1d); an approximately 6-fold decrease was observed in the diameter of the colonies (Supplementary Figure 1e). Additionally, the shUSP7-transfected T47D cell line (Supplementary Figure 1f) expressed a significantly decreased amount of USP7 (~5 fold) (Supplementary Figure 1g). The obtained CFU assay results in the T47D cell line (Supplementary Figure 1h) demonstrated that stable USP7 knockdown decreased colony numbers by 90% (Supplementary Figure 1i) and diameters by 50% (Supplementary Figure 1j). 

### 3.2. USP7 controls cell cycle 

**Figure 3:**
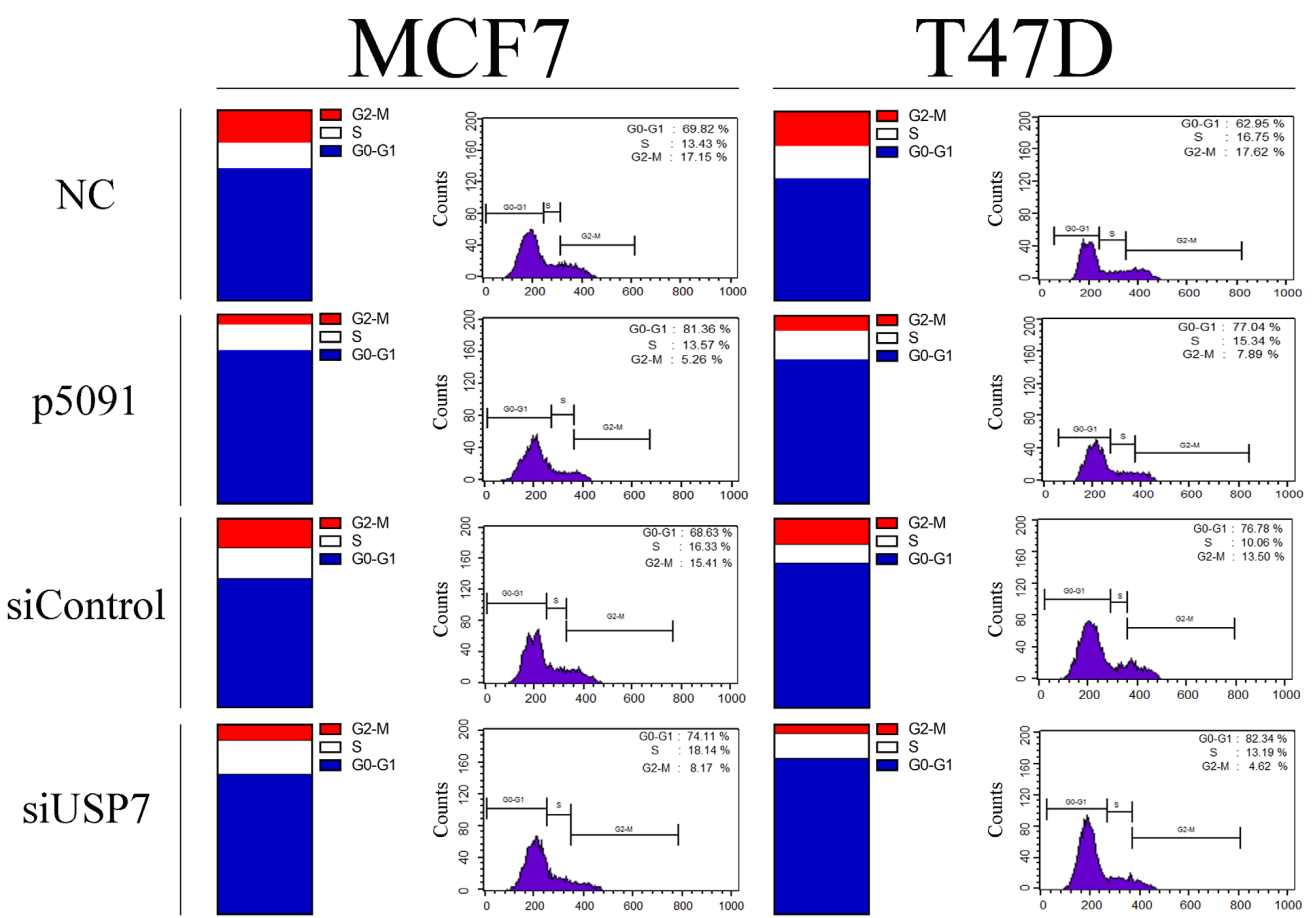
Allosteric inhibition and transient knockdown of USP7 alters cell cycle profile of MCF7 and T47D cells. MCF7 cells were treated with 10μM of p5091 for 2 days and T47D cells were treated with 10μM of p5091 for 72 h. Both of the cell lines were treated with 20 pmol siRNA for 48 h. NC: Negative control, siControl: Control small interfering RNA, siUSP7: USP7 small interfering RNA, number of replicates: 3.

USP7 inactivation caused alterations in cell cycle regulation of breast cancer cell lines. The obtained DNA histograms showed that MCF7 cells accumulate in G1 phase after p5091 treatment and the cell number difference between the control group and the p5091-treated group was calculated as 11.54%. In addition to this, after 10µM of p5091 treatment, 11.86% reduction was observed in the number of MCF7 cells that are in G2/M phase (Figure 3). Although no significant change was observed at S phase of the T47D cells, 14.45% difference was observed in accumulated cells in G1 phase after p5091 treatment. Cell cycle analysis also revealed that 17.62% of control T47D cells were in G2-M phase. In contrast, only 7.89% of p5091-treated T47D cells were in G2/M phase (Figure 3). 

USP7 knockdown by siRNA altered the cell cycle profile of MCF7 and T47D cell lines as well. The number of MCF7 cells in G1 phase was increased by 5.48%, and a 7.24% decrease was found in the number of the cells in G2/M phase after the USP7 level was reduced. However, no significant change was observed at S phase after siRNA application. Therefore, the decreased USP7 amount caused an arrest in G1 phase and a decrease in G2/M phase in MCF7 cells (Figure 3). A similar effect was also observed in T47D. G1 and S phases of T47D cells were expanded by 5.56% and 3.06% after siUSP7 application, respectively. Moreover, the number of the T47D cells in G2/M phase decreased by 8.88% after siUSP7 administration (Figure 3). Consequently, cell cycle results were found to be in agreement with the proliferation assay results. 

### 3.3. Cell migration capacity of breast cancer cells might be regulated by USP7

**Figure 4:**
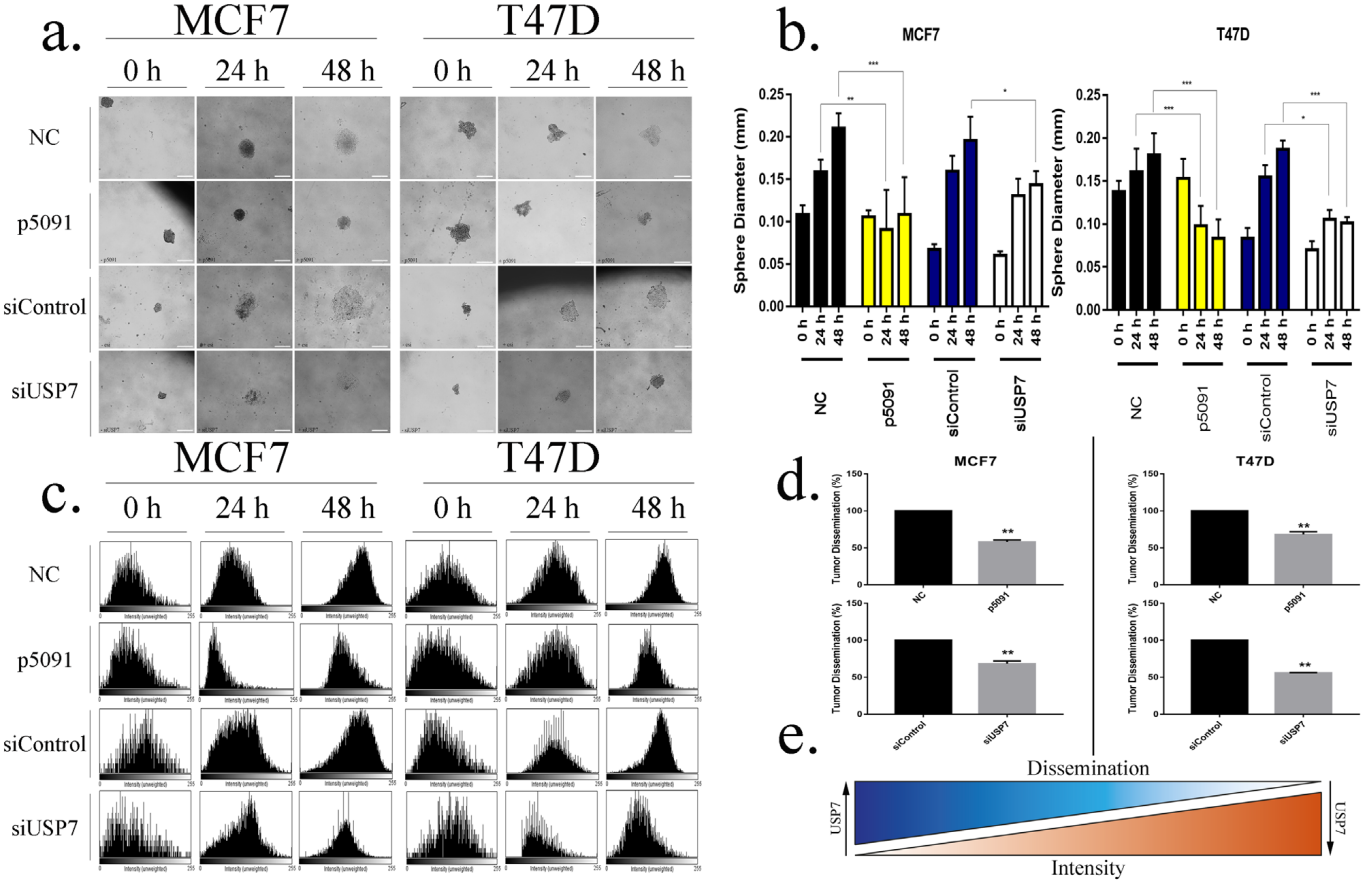
USP7 enzyme plays a critical role in tumor dissemination. (a) Microscopic images of tumor sphere dissemination profile after allosteric inhibition and transient knocking-down of USP7 in MCF7 and T47D cells. (b) Sphere diameter measurements of MCF7 and T47D cell lines after 10μM p5091 treatment (48 h for MCF7 and 72 h for T47D) and 2 days of siRNA (20 pmol) application. (c) Histogram representation of intensity measurements of MCF7 and T47D cells after 10μM p5091 administration (MCF7 for 48 h and T47D for 72 h) and 20 pmol of siRNA application for 2 days. (d) Tumor dissemination percentages of MCF7 and T47D cell lines after allosteric inhibition and transient knocking-down of USP7 enzyme. (e) Illustrated chart of the correlation between tumor sphere dissemination intensity and USP7. * P < 0.05, ** P < 0.005, *** P < 0.001, scale bar: 400μm (5x). NC: Negative control, siControl: Control s mall interfering RNA, siUSP7: USP7 small interfering RNA, number of replicates: 5.

The effect of USP7 downregulation on migratory phenotype was evaluated by sphere formation and dissemination analysis. The results demonstrated that dissemination of the p5091- and siRNA-treated spheres was decreased dramatically in both MCF7 and T47D cells (Figure 4a).

Inhibition of USP7 activity by p5091 and siRNA treatment diminished the migration capacity of both MCF7 and T47D cells, resulting in reduced sphere diameter after 24 h and 48 h compared to control. p5091-treated spheres were 37.5% smaller after 24 h in MCF7 cells and 45% smaller in T47D cells compared to baseline (Figure 4b). 

The difference between control and p5091 groups was more obvious after 48 h; 40% and 60% reduction in spheres size were observed in MCF7 and T47D cell cultures, respectively (Figure 4b). 

Although siRNA treatment did not cause a significant change in cell migration of MCF7 cells after 24 h, a 30% decrease in the diameter of siUSP7-treated spheres was observed after 48 h, indicating maximum gene downregulation at day 2 following siRNA treatment (Figure 4b). However, siUSP7 exerted 35% and 46% reduction at 24 h and 48 h respectively in T47D spheres (Figure 4b). 

Sphere dissemination of MCF7 and T47D cultures was determined via intensity measurements (Figure 4c). Less intensity is an indicator of cell migration on the tissue culture plate surface. Intensity analysis of cell lines was followed by the calculation of difference in sphere intensity, which was found to be negatively correlated with tumor dissemination for both T47D and MCF7 cell lines. USP7 protein inhibition by p5091 resulted in more intense spheres in both MCF7 and T47D cell lines even after 48 h of incubation. Similarly, knockdown of USP7 induced sphere intensity for both of the cell lines (Figure 4c). Correlatively, tumor sphere dissemination calculations of MCF7 demonstrated that USP7 inactivation decreased tumor dissemination by 42% after p5091 treatment and 32% after USP7 knockdown (Figure 4d). Similarly, siUSP7 application decreased dissemination by 44%, and p5091 treatment decreased tumor dissemination by 32% in T47D cells (Figure 4d). These results supported the negative correlation between tumor dissemination and intensity measurements (Figure 4e). In addition, scratch assay (in vitro cell migration) was performed to determine the effect of USP7 on cell migration (Supplementary Figure 2). The obtained results clearly highlight that deactivation of USP7 protein with its allosteric inhibitor p5091 decreased gap closure capability of MCF7 cells by 87.5%. Similarly, p5091 treatment decreased the gap closure capability of T47D cells by 95%. Additionally, compared to the control siRNA group, siUSP7 application also decreased the wound healing capacity of MCF7 and T47D cell lines by 64% and 66%, respectively (Supplementary Figure 2).

### 3.4. USP7 changes gene expression profile

**Figure 5:**
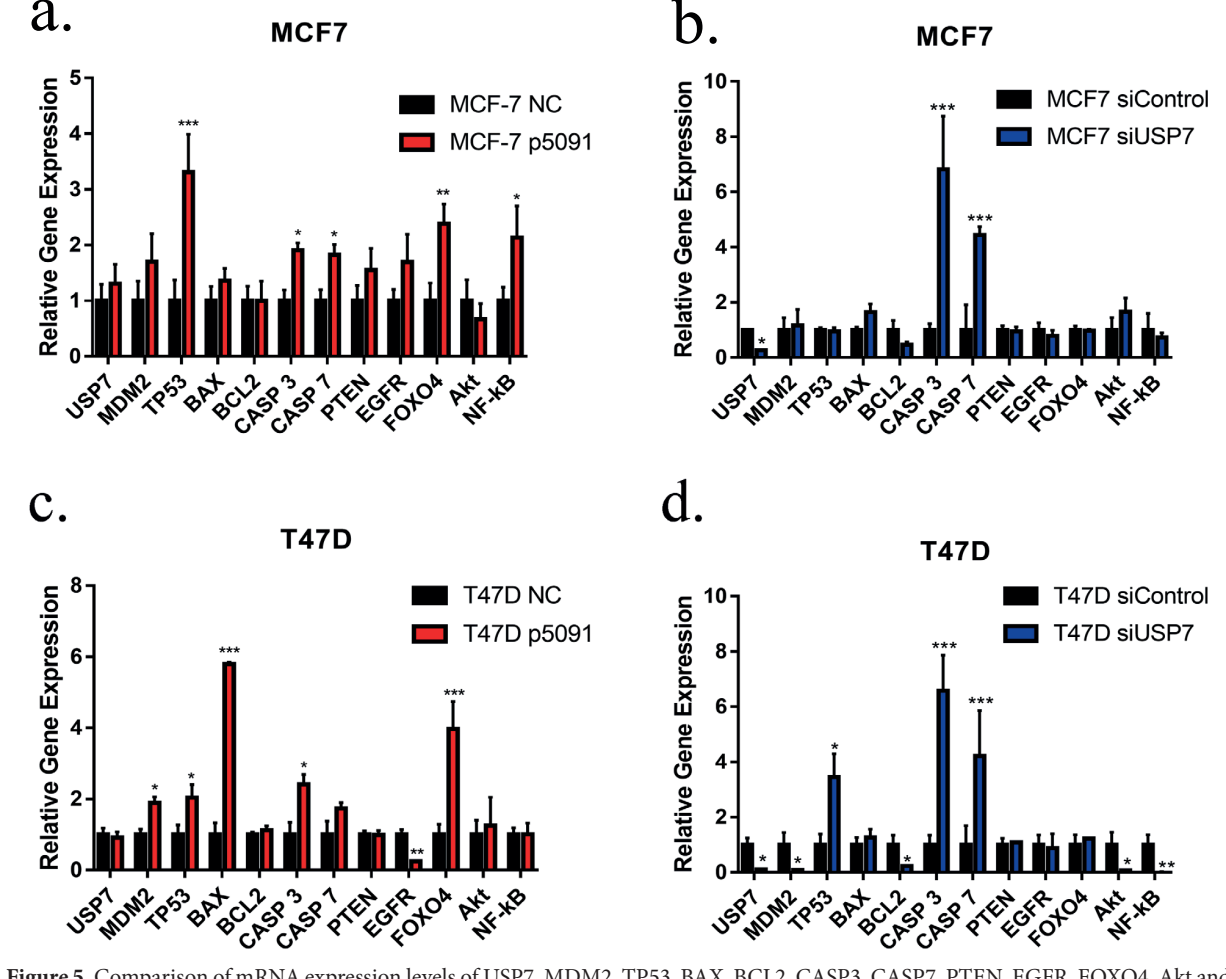
Comparison of mRNA expression levels of USP7, MDM2, TP53, BAX, BCL2, CASP3, CASP7, PTEN, EGFR, FOXO4, Akt and NF-κB in experimental groups. (a) Changes in gene expression profiles of MCF7 cell line after 2 days of p5091 (10μM) treatment and (b) 2 days of siRNA (20 pmol) administiration. (c) Alterations in gene expression profiles of T47D cell line after 3 days of p5091 (10μM) treatment and (d) 2 days of siRNA (20 pmol) administiration. * P < 0.05, ** P < 0.005, *** P < 0.001. NC: Negative control, siControl: Control s mall interfering RNA, siUSP7: USP7 small interfering RNA, USP7: Ubiquitin specific processing protease 7, MDM2: Mouse double minute 2 homolog, BAX: Bcl–2-associated X protein, BCL2: B-cell lymphoma 2, CASP3: Caspase 3, CASP7: Caspase7, PTEN: Phosphatase and tensin homolog, EGFR: Epidermal growth factor receptor, FOXO4: Forkhead box protein O4, Akt: Protein kinase B, NF-κB: Nuclear factor kappa B, number of replicates: 3.

qPCR experiments have clearly shown that p5091 treatment did not cause any significant change in USP7, MDM2, BAX, BCL2, PTEN, EGFR, and AKT gene expression levels in the MCF7 cell line. However, 3-fold upregulation of TP53 gene expression was observed as an apoptotic marker. CASP3 and CASP7 gene expression levels were increased by 1.9-fold and 1.8-fold respectively as late apoptosis indicators. An approximately 2-fold increase was observed for both FOXO4 and NF-kB mRNA levels (Figure 5a). 

siUSP7 treatment on MCF7 cells led to a dramatic decrease in USP7 gene expression (5-fold decrease) as expected. USP7 decrease had no noticeable effect on PTEN, FOXO4, TP53, MDM2, BAX, BCL2, PTEN, EGFR, FOXO4, AKT, and NF-kB genes which might be explained by the transient downregulation of the USP7 gene. On the other hand, CASP 3 and CASP 7 mRNA levels were found to be increased 6.8-fold and 4.4-fold, respectively, indicating that the cells were going through apoptosis (Figure 5b). 

p5091 treatment had a similar effect on the gene expression profile of T47D cells. USP7, BCL2, CASP7, and PTEN gene expressions did not change. Additionally, increased FOXO4 (3.9-fold), CASP 3 (≈2.5-fold), CASP 7 (≈2-fold), TP53 (2-fold), and MDM2 (1.8-fold) mRNA levels were observed as an indicator of apoptosis. p5091 caused a 5.7-fold increase in BAX, which is a proapoptotic gene (Figure 5c). siUSP7 treatment not only caused a decrease in UPS7 (10-fold), but also in reduced MDM2 (10-fold), BCL2 (5-fold), Akt (14.3-fold), and NF-kB (2.13-fold) gene expression in T47D cells. Similarly, increases in TP53 (3.4-fold), CASP3 (6.5-fold), and CASP 7 (4.2-fold) mRNA levels were observed as an indicator of apoptosis (Figure 5d).

## 4. Discussion

The ubiquitination pathway, as an important molecular mechanism for protein function, is involved in nearly every molecular process, such as the TP53-MDM2 pathway (Oren, 2003), which is crucial for apoptosis. Recent studies have focused on the inhibition of E3 ligases to rescue proteins from ubiquitination (Brahemi et al., 2010) and deubiquitination enzymes (DUBs) which are responsible for controlling the 26S proteasomal degradation (Amerik and Hochstrasser, 2004). Regulation of the ubiquitination process is mainly conducted by DUBs since not only do they deattach the ubiquitin tags from the protein, they also block the ubiquitination sides on proteins (Hoeller and Dikic, 2009). USP7, which is a member of the ubiquitin-specific protease deubiquitination family, is a unique example for the relevant regulation (Nijman et al., 2005). Thus, in the current study, we have aimed to identify USP7, which is a deubiquitination enzyme, as a new therapeutic target for breast cancer treatment regardless of TP3 mutation in vitro. Previous studies have demonstrated that p5091, which is a specific inhibitor of USP7, blocks cell proliferation of different types of cancers such as ovarian (Wang et al., 2017), prostate (Morra et al., 2017), and breast cancers (Xia at al., 2019). Our results show that suppression of USP7 activity via p5091 and siUSP7 caused a decrease in cell proliferation for both MCF7 and T47D cell lines regardless of their TP53 mutation status. CFU results also supported the cell proliferation data. Furthermore, the effect of p5091 on CFU was replicated with a colony-forming unit assay in which USP7 was knocked down stably, as there was no significant change in CFU capacity after transient knockdown of USP7.

Our results have shown that allosteric inhibition of USP7 did not change the USP7 level as expected (Carra et al., 2017), but enhanced the amount of the total ubiquitination in the cell, which confirms the deactivation of the USP7 enzyme. A decrease in USP7 enzyme activity altered the expression profiles of apoptotic genes as expected (Hu et al., 2019). Gene expression levels of TP53 and CASP3 in both MCF7 and T47D cell lines increased dramatically. It is known that the negative feedback mechanism of p53 protein is regulated by USP7 enzyme activity due to its interaction with Mdm2 protein (Wang 2019). Moreover, decreasing USP7 enzyme activity increases the self-ubiquitination capacity of the Mdm2 enzyme (Song et al., 2008). Thus, USP7 decrease resulted in noticeable upregulation of TP53 gene expression. A recent study has shown that inhibition of USP7 increases the Bax protein level in the MCF7 cell line, which harbors wild-type p53 protein (Xia at al., 2019). It is known that the activation of Bax protein can be controlled by p53 protein (Chen, 2016); the activated Bax protein increases apoptosis through the mitochondrial pathway (Borner and Andrews, 2014). Our results demonstrated that expression level of the BAX gene dramatically increased in the p53-mutated T47D cell line after inactivation of USP7; this increase may also support that the effect of USP7 inactivation is independent of p53.

Transient silencing of USP7 was found to be more efficient in overexpression of caspases 3 and 7, which are known as executioner caspases and are highly correlated with apoptosis (Liu et al., 2016). Surprisingly, USP7 knockdown decreases MDM2, AKT, and NF-κB gene expression levels in the T47D cell line, which harbors the p53 mutation. It is known that Mmd2 can be activated by Akt protein (Nakanishi et al., 2014) and that the activated Mdm2 is responsible for p53 degradation (Gupta et al., 2019). Additionally, it was found that p53 protein is suppressing NF-κB protein in lung cancer cell lines (Dey et al., 2007). However, our results show that USP7 knockdown decreases the expression levels of MDM2, AKT, and NF-κB genes without being dependent on p53 protein. 

Kessler et al. (2007) showed that knockdown of USP7 is responsible for some major alterations in the function of different proteins that participate in cellular events, including cell cycle regulation and DNA replication in colorectal adenocarcinoma. Furthermore, increased p21 level was observed after inactivation of USP7 by p5091 in ovarian cancer (Wang et al., 2017). In the current study, remarkable cell accumulation at the G0–G1 phases was observed after USP7 inactivation in MCF7 and T47D cell lines. This accumulation is in positive correlation with the literature showing that USP7 inhibition causes a G0–G1 arrest in breast cancer cell lines (Xia at al., 2019). 

 Three-dimensional tumor sphere formation, cell surface attachment, and migration of cells from the sphere to the surrounding extracellular matrix are used to mimic carcinogenic phenotype, metastasis, and invasion (Weiswald et al., 2015; Hosseini et al., 2016). Moreover, alterations in sphere intensity were measured daily as migrated spheres become less intense. Inhibition of USP7 via p5091 administration or downregulation by siUSP7 siRNA disrupted sphere integrity; it inhibited cell migration from spheres and as a result increased the sphere intensity.

To assess whether proliferative features of cancer cells and metastatic potential could be actually decreased by a differential amount of USP7, scratch assay was performed. It is indicated in the literature that MCF7 and T47D cells migrate less to the wounded area after silencing the several factors that have positive effects on cell proliferation (Yoshimura et al., 2016). Both MCF7 and T47D cell lines migrated to the scratched area significantly less after inhibition of USP7 enzyme activity by p5091 or siUSP7. Therefore, it has been proven that USP7 enzyme activity is necessary for cellular metastasis. Although an indirect correlation between USP7 and the metastasis of gastric cancer has been shown previously by altering the levels of USP7 mediated histone demethylase PHF8 (Li et al., 2017), our study demonstrated the direct correlation between USP7 enzyme activity and cancer cell migration ability in a p53-independent manner for the first time. 

## 5. Conclusion

Overall, USP7 as a deubiquitination enzyme might be a molecular regulator in breast cancer and could be a target for future gene-editing strategies. Downregulation of USP7 activity, which resulted in a significant increase of apoptosis-related genes, blockage of cell proliferation, and reduced cell migration, showed remarkable anticancer activity in both MCF7 and T47D cell lines. The potential anticancer activity of USP7 was shown to be p53-independent in vitro; it should be studied in detail by RNA seq transcriptomic analysis in various breast cancer and healthy breast epithelium cell types. Identification of USP7 as a therapeutic target might be possible through detailed gene editing analysis on more invasive breast cancer cell lines and healthy breast epithelial cell lines. In vivo tumor formation and gene therapy strategies are necessary to understand the exact molecular mechanism underlying the regulatory role of USP7 on tumor formation and progression.

## Acknowledgments

The study received grants from Yeditepe University. The authors also acknowledge Ayla Burçin Asutay for her assistance in flow cytometry analysis.

Supplementary Materials
