# Supplementary material for: Ubiquitin-specific protease 7 downregulation suppresses breast cancer in vitro
Source: Turk J Biol. 2020 Aug 19;44(4):145–57. doi: 10.3906/biy-1912-83 (PMC7478133; doi:10.3906/biy-1912-83)
Supplement: Supplementary file 1 — Supplementary Materials [file turkjbio-44-145-sup001.pdf]

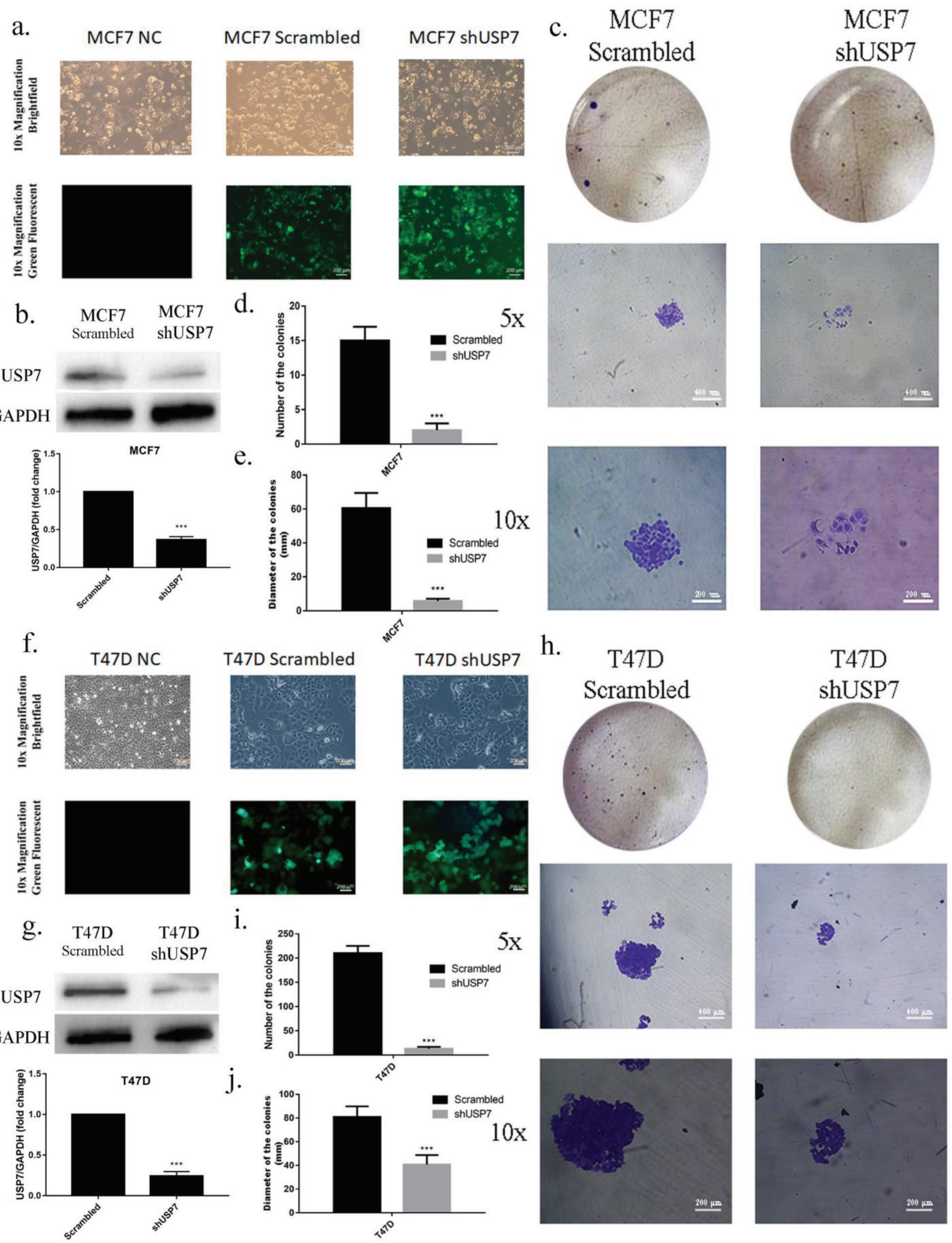

Supplementary Figure 1. Stable knockdown of USP7 blocks CFU formation in vitro. (a) Fluorescent and bright filtered images of MCF7 after lentiviral infection. Both scrambled and shUSP7 plasmids have green fluorescent protein genes. (b) Total USP7 protein levels of MCF7 cell line after targeting USP7 protein by lentiviral shRNA vector. GAPDH antibody was used as a loading control. (c) Microscopic images of CFU assay after knocking down USP7 in MCF7 cells. (d) Number of the colonies and (e) diameter of the colonies after USP7 knock down in MCF7 cells. (f) Fluorescent and bright filtered images of T47D after lentiviral infection with scrambled and shUSP7 plasmids which both have green fluorescent protein genes. (g) Total USP7 protein levels of T47D cell line after targeting USP7 protein by lentiviral shRNA vector. GAPDH antibody was used as a loading control. (h) Microscopic images of CFU assay after knocking down USP7 in T47D cells. (i) Number of the colonies and (j) diameter of the colonies after knocking down USP7 in T47D cells. *P < 0.05, ***P < 0.001, scale bar: 400µm (5x), 200µm (10x). NC: Negative Control, shUSP7: USP7 specific short hairpin RNA, number of replicates: 3.

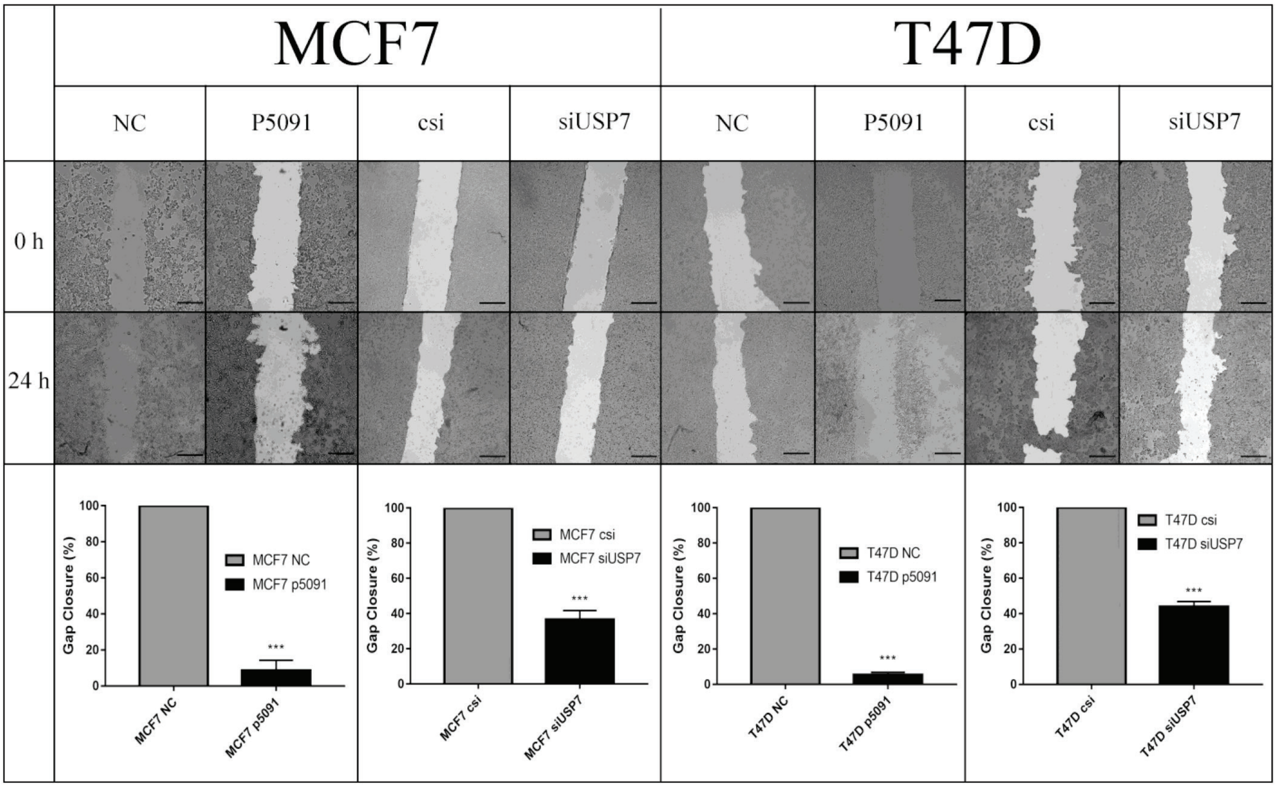

Supplementary Figure 2. Allosteric inhibition and transient knockdown of USP7 decreases gap closure percentages thus migration capacity of MCF7 and T47D cells. 10µM of p5091 and 2 days siRNA treatment were applied. ***P < 0.001, scale bar: 200µm. NC: Negative control, siControl: Control small interfering RNA, siUSP7: USP7 small interfering RNA, number of replicates: 3.
